# Optimizing Skeletal Muscle Anabolic Response to Resistance Training in Aging

**DOI:** 10.3389/fphys.2020.00874

**Published:** 2020-07-23

**Authors:** Yori Endo, Atousa Nourmahnad, Indranil Sinha

**Affiliations:** ^1^Division of Plastic Surgery, Brigham and Women’s Hospital, Harvard Medical School, Boston, MA, United States; ^2^Harvard Department of Stem Cell and Regenerative Biology, Harvard Stem Cell Institute, Cambridge, MA, United States

**Keywords:** sarcopenia, resistance training, skeletal muscle hypertrophy, exercise, aging

## Abstract

Loss of muscle mass and strength with aging, also termed sarcopenia, results in a loss of mobility and independence. Exercise, particularly resistance training, has proven to be beneficial in counteracting the aging-associated loss of skeletal muscle mass and function. However, the anabolic response to exercise in old age is not as robust, with blunted improvements in muscle size, strength, and function in comparison to younger individuals. This review provides an overview of several physiological changes which may contribute to age-related loss of muscle mass and decreased anabolism in response to resistance training in the elderly. Additionally, the following supplemental therapies with potential to synergize with resistance training to increase muscle mass are discussed: nutrition, creatine, anti-inflammatory drugs, testosterone, and growth hormone (GH). Although these interventions hold some promise, further research is necessary to optimize the response to exercise in elderly patients.

## Introduction

The loss of skeletal muscle mass with aging is a well-known phenomenon (Doherty, 2003). Lean muscle mass decreases substantially after the age of 60 (Melton et al., 2000). Severe, aging-associated loss of muscle mass and strength, also termed sarcopenia (Rosenberg, 1989) and dynapenia (Volpi et al., 2004), respectively, have profound consequences that extend beyond simple loss of mobility (Wolfson et al., 1995). Specific diagnostic criteria for sarcopenia continues to evolve (Cruz-Jentoft et al., 2010; Studenski et al., 2014), but it manifests with increased insulin resistance, loss of bone density, and an increase in falls (Dutta and Hadley, 1995; Rantanen et al., 1999). As such, these patients are at an increased risk of all-cause mortality, incident and mobility disabilities, and loss of independence (Angulo et al., 2020). From a public health perspective, the economic burden of caring for sarcopenic patients is tremendous and accounts for nearly $28.5 billion per year in expenditures, after adjusting for inflation (Janssen et al., 2004).

Many recent studies suggest that regimented physical activity, including resistance training, can be beneficial in maintaining muscle strength and function in elderly individuals (Pahor et al., 2014; Losa-Reyna et al., 2019; Martínez-Velilla et al., 2019; Rodriguez-Mañas et al., 2019; Yu et al., 2019). However, although physical training is beneficial at any age, the anabolic response to exercise decreases substantially with aging (Welle et al., 1996; Phillips et al., 2017; Lee et al., 2019). This review explores the mechanisms of cellular and molecular adaptations of skeletal muscle to exercise, with a focus on the aging-associated changes that cause hinderance of its anabolic response to exercise. We further evaluate the efficacy of supplements commonly used with physical training to optimize the exercise benefit on skeletal muscle, with the ultimate goal of preventing sarcopenia and associated adverse events.

## Skeletal Muscle and Aging

Aging is associated with changes in multiple biological processes and impacts nearly every facet of tissue homeostasis (López-Otín et al., 2013). Changes specific to skeletal muscle include diminished fiber number and cross-sectional area (Lexell et al., 1988), a decline in fast-twitch muscle-fibers (Lexell, 1995), and increased fat infiltration (Marcus et al., 2010). These structural changes are responsible for the loss of strength that accompanies muscle aging (Thompson, 2002; Distefano and Goodpaster, 2018). A complex network of signaling factors are precisely regulated to maintain myogenesis and muscle mass. Protein metabolism is regulated by Akt and mammalian target of rapamycin (mTOR) signaling pathways, which are, in turn, activated by various anabolic stimuli to bring about hypertrophic response in skeletal muscle. The induction of the insulin-like growth factor (IGF) pathway, upstream of Akt/mTOR prevents muscle atrophy (Yoon, 2017), highlighting its importance in the maintenance of muscle mass. The circulating levels of IGF-1 and IGF-1 binding proteins are decreased in aging, with a corollary reduction in mTOR activation in sarcopenic individuals (Pallafacchina et al., 2002; Léger et al., 2008; Deane et al., 2013; Sharples et al., 2013).

The maintenance of muscle mass may be further limited by diminished nutritional stimuli due to poor nutritional intake (Buford et al., 2010). A lack of nutrients, including essential amino acids (EAAs), is further paired with improper post-prandial nutrient handling in the elderly (Wall et al., 2015). These changes contribute to an inability to increase muscle protein synthesis in response to exercise or nutritional availability and results in muscle atrophy with aging (Wilkinson et al., 2018). The inability to properly utilize nutrition is synonymous with anabolic resistance, in which skeletal muscle in old age cannot gain mass despite appropriate cues. Two major factors contributing to this phenomenon in elderly subjects are poor nutrition and a reduction in regimented physical activity (Steffl et al., 2017; Wilkinson et al., 2018).

Muscle hypertrophy can also be negatively regulated by catabolic signals, most prominent of which are the transforming growth factor (TGF)-β superfamily and related cytokines. Myostatin and TGF-β both limit muscle hypertrophy by regulating the expression of genes involved in differentiation and proliferation in muscle stem cells (Langley et al., 2002; Yang et al., 2007), increasing protein degradation (Sartori et al., 2009), and inhibiting mTOR activation by anabolic stimuli in mature myofibers (Trendelenburg et al., 2009). While their role in sarcopenia remains unclear, some studies have shown that an age-related increase in TGF-β signaling from myofibers occurs in parallel with a decline in Notch signaling in satellite cells (Conboy et al., 2003; Chakkalakal et al., 2012), thus resulting in a reduced regenerative capacity of aged muscle. Myostatin was found to be increased in type II muscle fibers (Shibaguchi et al., 2018), suggesting that myostatin may play a role in selective type II fiber atrophy as seen in old age.

Androgenic depravation may be a factor contributing to sarcopenia in old males (Katznelson et al., 1996; Kenny et al., 2001; Ly et al., 2001). Testosterone promotes skeletal muscle hypertrophy directly by increasing protein synthesis (Ferrando et al., 1998) and muscle stem cell division (Powers and Florini, 1975), and indirectly by increasing IGF-1 expression via ERK and mTOR signaling (Sculthorpe et al., 2012). The exact role of testosterone in sarcopenia remains to be established; however, a study reported a significant association between serum-free testosterone and muscle mass in well-nourished, elderly men (Baumgartner et al., 1999). A corollary study demonstrated that lower circulating testosterone was associated with decreased maximal performance capacity in elderly men (Häkkinen and Pakarinen, 1993).

Chronic inflammation, as occurs with aging, has been shown to have detrimental effects on muscle physiology. In particular, the NF-κB pathway may be causative in limiting skeletal muscle repair following injury and hastening atrophy (Li et al., 2008). NF-κB is highly expressed in elderly people with muscle wasting (Bruunsgaard and Pedersen, 2003) and its level correlates with decreased anabolic response (Cuthbertson et al., 2005). In multiple preclinical models, NF-κB limited myoblast differentiation and regeneration following injury (Oh et al., 2016). Taken together, the evidence suggests that pharmacological inhibition of chronically activated NF-κB may limit aging-associated muscle loss. Indeed, non-steroidal anti-inflammatory drugs (NSAID)s promote muscle regeneration following injury, although its benefit in limiting sarcopenia remains to be elucidated (Thaloor et al., 1999; Oh et al., 2016).

In addition to alterations in the systemic milieu, intrinsic changes within myofibers and muscle stem cells with aging also affects the ability of skeletal muscle to respond to anabolic stimuli. Hyperphosphorylation of mTORC1, which impairs its activation (Kang et al., 2013), is found in aged muscle of human (Markofski et al., 2015). Therefore, defective mTOR signaling likely underlies the resistance of skeletal muscle to anabolic stimuli (Guillet et al., 2004), insulin resistance (Rasmussen et al., 2006), and impaired protein/glucose homeostasis in aged skeletal muscle (Petersen et al., 2015). Mitochondrial dysfunction has also been associated with sarcopenia (Coen et al., 2013) and mitochondrial DNA damage has been shown to cause muscle wasting (Amara et al., 2007). While there are no pharmacotherapeutics that are efficacious in attenuating skeletal muscle loss in aging, resistance training may limit some of these pathologic aging associated changes in skeletal muscle by augmenting mTOR activity (Song et al., 2017).

## Effect of Resistance Exercise Training on Skeletal Muscle

Physical activity, especially resistance training, is unequivocally beneficial for elderly patients with regards to enhancing muscle mass and strength (Fiatarone et al., 1990; Dibble et al., 2006; Peterson et al., 2011; Drummond et al., 2012). A recent review found that when progressive resistance training (PRT) is performed 2–3 times a week at a high intensity, it results in improved physical function and strength (Liu and Latham, 2009). The frequency and duration of resistance exercise in elderly are recommended at 2–4 times per week on alternating days and lasting 30–60 min each; 1–3 sets of 8–15 reps at 80% of one-rep maximum strength, with a monthly progressive adjustment (American College of Sports Medicine Position Stand, 1998; Law et al., 2016). In skeletal muscle, functional overload induces hypertrophy resulting in increased muscle mass and fiber size in a dose-dependent manner (Frontera et al., 1988). Previous studies have demonstrated that resistance exercise or muscle contraction increases overall muscle protein turnover in favor of protein synthesis through the activation of the mTOR pathway (Biolo et al., 1995). In addition to its direct anabolic effect, exercise has been shown to increase the circulating levels of IGF-1 (Borst et al., 2001) and androgens (Hawkins et al., 2008), while decreasing myostatin levels (Hittel et al., 2010). Furthermore, physical activity promotes restoration of insulin sensitivity, mitochondrial biogenesis, and reduces inflammation (Nieman et al., 2003; Campbell and Turner, 2018).

## Attenuated Exercise Benefit in the Elderly on Muscle Mass and Strength

Despite the consensus that regimented physical training is beneficial for the maintenance of strength and function, numerous studies suggest that the effects of exercise on skeletal muscle physiology decreases with aging. Anabolic resistance describes the inability of the body to add muscle mass despite physical activity (Kumar et al., 2009; Rivas et al., 2012; Francaux et al., 2016). In older patients, the increase in lean muscle mass following resistance exercise training is substantially less than younger subjects (Pedersen et al., 2003). As such, the gain in strength following regimented exercise programs are substantially less in the elderly (Welle et al., 1996; Kosek et al., 2006; Booth and Laye, 2010). Diminished induction of muscle regeneration following exercise further dampens the overall hypertrophic response in the elderly (Ogawa et al., 1992; Behnke et al., 2012; Suetta et al., 2013). In addition, elderly patients suffer from impaired muscle activation secondary to aging-associated changes in motor unit density and morphology (Campbell et al., 1973; Raj et al., 2010; Hepple and Rice, 2016). Resistance exercise training improves innervation and thus muscle strength in elderly even without fiber hypertrophy (Messi et al., 2016). Despite exercise, however, the numbers of motor units may still decline with aging (Power et al., 2012; Piasecki et al., 2016), limiting the functional improvement attainable from exercise. Understanding the limitations of resistance training and potential mechanisms underlying this phenomenon is critical for improving exercise benefit in the elderly population.

## Optimizing Skeletal Muscle Response to Exercise in Aging

### Nutritional Supplementation

The acute anabolic responses to feeding and exercise were found to be dampened in old subjects compared to their young counterparts, thus limiting their recovery, and muscle growth (Cuthbertson et al., 2005; Durham et al., 2010). It has been hypothesized that the blunted increase in protein synthesis following acute muscle loading may influence the smaller gains in lean tissue following resistance exercise training in older adults (Durham et al., 2010). As such, supplementation of high-quality protein may improve anabolic response to a single bout of exercise (Drummond et al., 2008; Dideriksen et al., 2011; Pennings et al., 2011). Whole protein supplements such as whey and casein, both milk-derivatives, are popularly ingested with the intention to increase muscle mass. Casein, when used as a pre-sleep protein supplement, has been shown to increase myofibrillar protein synthesis rates overnight in older adults (Kouw et al., 2017). When combined with a bout of resistance exercise in the evening, rates of protein synthesis were even higher (Holwerda et al., 2016). While fiber hypertrophy was seen with pre-sleep protein ingestion during a resistance training regimen in young men (Snijders et al., 2015), outcomes in older individuals require further investigation (Holwerda et al., 2016).

Specific amino acid supplements are also available, in the forms of EAAs, branched-chain amino acids (BCAAs), and leucine. Leucine-rich EAA supplementation enhanced muscle strength following exercise, although the study included elderly women only (Kim et al., 2012). It is important to note, however, that prolonged protein supplementation with whey or casein, in the setting of a training program, does not appear to improve the exercise response in elderly patients (Godard et al., 2002; Kukuljan et al., 2009; Verdijk et al., 2009). β-hydroxy-β-methylbutyrate (HMB), a metabolite of leucine which directly activates mTOR, has also been investigated and increased lean muscle mass and strength in sarcopenic individuals (Oktaviana et al., 2019). In total, protein and amino acids are a promising exercise supplement for the elderly. Current recommendations for daily protein intake in most older individuals are 1.2–1.5 grams protein/kilogram body weight (Duetz et al., 2014). Interventional trials are required to identify the appropriate composition of proteins and/or amino acids, as well as the timing of delivery.

Separately, creatine is essential for muscle ATP production and has been commonly ingested to enhance anabolic response to exercise. Multiple studies have presented some evidence that creatine treatment, in combination with resistance training, enhances gains in muscle mass and strength following exercise beyond what is attainable with resistance exercise alone (Candow et al., 2019). The benefit of creatine therapy alone without resistance training remains unclear; some have suggested that creatine ingestion improves lean muscle mass in the elderly (Gotshalk et al., 2002), whereas others have observed no benefit in muscle mass or strength with creatine administration (Lobo et al., 2015; Baker et al., 2016; Chami and Candow, 2019). However, in elderly subjects, supplementing resistance training with creatine increased lean muscle mass and strength when compared to placebo (Candow et al., 2014; Devries and Phillips, 2014; Chilibeck et al., 2017). In addition to its known role in ATP production, numerous studies suggest that creatine’s positive effect on aging muscle may work through several mechanisms, including by inducing proteins downstream of the mTOR pathway (Safdar et al., 2008), decreasing protein degradation (Parise et al., 2001), and functioning as an antioxidant (Sestili et al., 2011). Importantly, creatine therapy appears to have a low risk profile with minimal adverse effects (Kreider et al., 2017), making it an attractive supplement.

Other recently proposed nutritional supplements to counter sarcopenia and dynapenia include vitamin D and omega-3 polyunsaturated fatty acids. Vitamin D is diminished by up to 4 fold in older adults (MacLaughlin and Holick, 1985). Low vitamin D levels have been linked to muscle atrophy (Visser et al., 2003). Several studies found that vitamin D3 supplementation in the elderly results in increased muscle strength (Moreira-Pfrimer et al., 2009) as well as reduction in falls and fractures when combined with calcium (Pfeifer et al., 2009). However, others have reported no improvement in functional capacity with vitamin D supplementation (Uusi et al., 2015; Levis and Gómez-Marín, 2017; Shea et al., 2019). Omega-3, commonly found in fatty fish and seafood, may also limit sarcopenia progression and improve protein synthesis in response to anabolic stimuli (Smith et al., 2011). In addition, multiple studies show that omega-3 augments the effects of resistance training and further increases muscle mass and strength in older adults (Rodacki et al., 2012; Da Boit et al., 2017). Further interventional studies will be required to better define the efficacy and dosage for these compounds, but both are potentially efficacious supplements.

Multi-ingredient protein (MIP)-based supplements may prove to be more efficacious in improving muscle mass and strength gains with exercise as compared to single nutritional supplements alone. In a clinical trial, a MIP supplement consisting of whey protein, creatine, calcium, vitamin D, eicosapentaenoic acid, and docosahexanoic acid improved both lean muscle mass and strength in elderly patients, during exercise, as compared to placebo (Bell et al., 2017; O’Bryan et al., 2020). However, within a metanalysis, there was no benefit in muscle strength and mass, as compared to protein supplementation alone, in response to exercise (O’Bryan et al., 2020). This highlights that future research must focus on defining specific combinations and dosages.

### NSAID Therapy

Chronic, age-related inflammation in skeletal muscle may play a role in aging-associated muscle loss (Barnes and Karin, 1997). As mentioned previously, NF-κB, a master transcriptional regulator of inflammation, becomes upregulated in skeletal muscle with aging (Hayden and Ghosh, 2004). This has led to investigations of whether NF-κB inhibition using commercially available NSAIDs can improve the maintenance of muscle mass (Yamamoto and Gaynor, 2001). Inhibition of NF-κB directly improves muscle regeneration after injury in aged muscle (Oh et al., 2016) and limits muscle atrophy by decreasing MuRF signaling (Cai et al., 2004). The efficacy of NF-κB inhibition, using commercially available NSAIDs, on the maintenance of muscle mass and strength in response to exercise has been explored in many clinical studies in elderly patients. A 3-month bout of resistance exercise in elderly patients with knee osteoarthritis, NSAIDs therapy resulted in a mild improvement in muscle strength, however, without hypertrophy (Petersen et al., 2011). Other studies found that NSAID treatment augmented training-induced improvement in strength with associated muscle hypertrophy and limited muscle catabolism (Trappe et al., 2011, 2013). Others have instead shown that NSAID supplementation does not improve skeletal muscle strength or function during physical training (Dideriksen et al., 2016). In addition, it should be noted that NSAID therapy is not without its risks in the elderly population. Chronic NSAID use can result in risk of renal failure, cardiovascular events, and gastrointestinal ulceration (Wongrakpanich et al., 2018). As such, the use of NSAIDs during exercise remains a controversial, but potential treatment to augment response to exercise in the aging population. Improved specificity and identifying the correct dosage are, however, requisite to further promotion of this therapy.

### Testosterone Therapy

Testosterone has emerged as another potential supplement to exercise for the elderly population. Multiple studies have demonstrated that testosterone levels decrease with age (Morley et al., 1997; Wang et al., 2009). Testosterone administration to elderly patients increases both muscle mass and maximal voluntary strength in a dose-dependent fashion, possibly by the induction of myogenic gene expression (Bhasin et al., 2001). Despite this assertion, the additional benefits of physiological testosterone replacement in elderly patients remains unclear. A prospective study demonstrated increased upper body strength following testosterone treatment of elderly patients with low to normal serum testosterone, but this treatment did not offer any benefit beyond resistance exercise alone (Hildreth et al., 2013). Others have similarly reported no synergistic or additional benefits of testosterone treatment in PRT (Sullivan et al., 2005). Of note, this is in direct contrast to the benefits of supra-physiological testosterone supplementation with regards to muscle strength and mass in young men, in whom combined treatment with testosterone and exercise was more efficacious than treatment with testosterone or exercise alone (Bhasin et al., 1996). Therefore, it is necessary to consider adjustment of the duration and dosage of testosterone supplementation in exercise regimens for the elderly before conclusion about its efficacy can be drawn. Additionally, like NSAID therapy, testosterone supplementation does not come without potential adverse events, and therefore the clinical efficacy of testosterone for sarcopenia treatment should be carefully evaluated (Basaria et al., 2010).

### Growth Hormone/Insulin-Like Growth Factor Supplementation

The growth hormone (GH) axis is another area that has received attention as a potential supplement for exercise therapy for the elderly. GH is made in the pituitary gland and promotes IGF-1 (insulin growth factor) expression in skeletal muscle (Jorgensen et al., 2006; Velloso, 2008). IGF-1, in turn, stimulates the Akt/mTOR pathway which, as discussed before, promotes muscle anabolism and protein synthesis in response to exercise (Bolster et al., 2003). In elderly patients, GH treatment increases lean body mass and decreases fat-to-muscle ratio from baseline, although it is unclear as to whether this was attributable to increased skeletal muscle mass (Rudman et al., 1990, 1991). However, multiple studies have shown that healthy elderly patients do not see any additional benefit in strength or muscle hypertrophy with GH supplementation as compared to exercise alone, even at 6-month follow-up (Taaffe et al., 1994, 1996; Hennessey et al., 2001; Lange et al., 2002), despite confirmation of increased levels of circulating IGF-1. Interestingly, IGF-1 administration in isolation does not increase lean muscle mass. Its effects in combination with exercise, however, have not been independently tested (Friedlander et al., 2001). Separately, losartan, an angiotensin II type I receptor blocker which potentiates IGF-1 activity, failed to improve the anabolic response to physical resistance training (Heisterberg et al., 2018). Despite the integral role of the GH/IGF axis on muscle development or hypertrophy, it does not appear to have a therapeutic benefit in physical training in healthy individuals.

## Conclusion

Aging is a complex and heterogenous process. It is, however, uniformly associated with loss of skeletal muscle mass, strength, and function. Resistance exercise in older patients unequivocally results in substantial benefits exemplified by muscle fiber hypertrophy, increased strength, extended independent living, and reduced fall risk (Fragala et al., 2019). Many efforts have focused on improving this response further with nutritional supplements, anti-inflammatory drugs, and anabolic agents. While numerous studies have reported synergistic benefits of combining a supplement with exercise, many others suggest marginal benefits versus exercise alone, especially in elderly individuals. Future studies should utilize a standard resistance training regimen, guided by previously published position statements (American College of Sports Medicine Position Stand, 1998), with resistance exercise three times weekly at 30 min per bout at 60–80% resistance. Moreover, supplements must be individualized to patients. Elderly patients with low levels of IGF-1 or testosterone may benefit from those specific supplements, where as other elderly patients may not. New studies may focus on MIP therapies combining supplements which have demonstrated significant benefit with regards to muscle mass and strength during exercise, such as combining EAAs, Creatinine, Vitamin D, and omega-3 fatty acids. Heterogeneity in the patient population, physical training intensity, and duration of interventions make it difficult to draw generalizable conclusions, but understanding the mechanisms of anabolic resistance and augmenting response to exercise is paramount to maintaining muscle strength and function in aging.

## Author Contributions

All authors listed have made a substantial, direct and intellectual contribution to the work, and approved it for publication.

## Conflict of Interest

The authors declare that the research was conducted in the absence of any commercial or financial relationships that could be construed as a potential conflict of interest.
